# Glycosylation of Immunoglobulin G: Role of Genetic and Epigenetic Influences

**DOI:** 10.1371/journal.pone.0082558

**Published:** 2013-12-06

**Authors:** Cristina Menni, Toma Keser, Massimo Mangino, Jordana T. Bell, Idil Erte, Irena Akmačić, Frano Vučković, Maja Pučić Baković, Olga Gornik, Mark I. McCarthy, Vlatka Zoldoš, Tim D. Spector, Gordan Lauc, Ana M. Valdes

**Affiliations:** 1 Department of Twins Research and Genetic Epidemiology, Kings College London, London, United Kingdom; 2 Faculty of Pharmacy and Biochemistry, University of Zagreb, Zagreb, Croatia; 3 Glycobiology Laboratory, Genos, Zagreb, Croatia; 4 Oxford Centre for Diabetes, Endocrinology & Metabolism, University of Oxford, Oxford, United Kingdom; 5 Wellcome Trust Centre for Human Genetics, University of Oxford, Oxford, United Kingdom; 6 Faculty of Science, University of Zagreb, Zagreb, Croatia; 7 Academic Rheumatology, University of Nottingham, Nottingham, United Kingdom; The Scripps Research Institute and Sorrento Therapeutics, Inc., United States of America

## Abstract

**Objective:**

To determine the extent to which genetic and epigenetic factors contribute to variations in glycosylation of immunoglobulin G (IgG) in humans.

**Methods:**

76  *N*-glycan traits in circulating IgG were analyzed by UPLC in 220 monozygotic and 310 dizygotic twin pairs from TwinsUK. A classical twin study design was used to derive the additive genetic, common and unique environmental components defining the variance in these traits. Epigenome-wide association analysis was performed using the Illumina 27k chip.

**Results:**

51 of the 76 glycan traits studied have an additive genetic component (heritability, *h* ^*2*^)≥  0.5. In contrast, 12 glycan traits had a low genetic contribution (h^2^<0.35). We then tested for association between methylation levels and glycan levels (*P*<2 x10^-6^). Among glycan traits with low heritability probe cg08392591 maps to a CpG island 5’ from the *ANKRD11* gene, a p53 activator on chromosome 16. Probe cg26991199 maps to the *SRSF10* gene involved in regulation of RNA splicing and particularly in regulation of splicing of mRNA precursors upon heat shock. Among those with high heritability we found cg13782134 (mapping to the *NRN1L* gene) and cg16029957 mapping near the *QPCT* gene to be array-wide significant. The proportion of array-wide epigenetic associations was significantly larger (*P*<0.005) among glycans with low heritability (42%) than in those with high heritability (6.2%).

**Conclusions:**

Glycome analyses might provide a useful integration of genetic and non-genetic factors to further our understanding of the role of glycosylation in both normal physiology and disease.

## Introduction

Glycans constitute the most abundant and diverse form of the post-translational modifications. All cell surface and secreted glycoproteins that contain appropriate sequences (Asn-X-Ser/Thr where X is any amino acid except proline) can potentially acquire N-linked oligosaccharides (N-glycans) while they travel through the endoplasmic reticulum and the Golgi compartments [1]. Glycans can influence disease development in many syndromes such as congenital disorders of glycosylation, cancer, rheumatoid arthritis and AIDS [2]. Glycans are crucial for the immune system, as some of the most important interactions between the immune system and viruses and bacteria are mediated by protein-glycan interactions. Moreover, glycans are key in the recognition of non-self events and an altered glycome can lead to autoimmune disorders [3]. The biological functions of glycans go from basic structural roles to development, protein folding and immune response. Glycosylation is known to be affected by factors such as sugar nucleotide concentration, type of glyco-enzymes and their expression levels [1].

While genes unequivocally determine the structure of each polypeptide, there is no genetic template for the glycan part [4]. Instead, hundreds of genes and their products interact in the complex pathway of glycan biosynthesis resulting in a very complex biosynthetic pathway that is further complicated by both direct environmental influence (nutrition, hormonal status, etc) and epigenetic memory of past environmental effects (altered gene expression) [5-8]. 

Some recent studies [9,10] demonstrated large variability of plasma proteins and immunoglobulin G (IgG) *N*-glycome composition in a population. However, longitudinal studies in individuals also revealed very high temporal stability of the individual plasma glycome [11] indicating stable long-term regulation of the glycosylation machinery. 

Several studies have already shown that there are consistent genetic factors that affect circulating levels of some IgG *N*-glycans [12-14]. However, the extent to which genetic and non-genetic factors affect glycan levels is still unexplored. 

In this study we aim to determine the extent to which individual differences in IgG glycosylation pattern reflect genetic *versus* environmental influences by estimating heritability in a cohort of female twins. We, then, hypothesized that IgG glycosylation pattern with low heritability (h^2^≤0.35) might be epigenetically mediated and we explored the relationship with epigenetic data.

## Results

The study cohort comprised 220 monozygotic (MZ) and 310 dizygotic (DZ) twin pairs, and the baseline characteristics are presented in Table **1**. MZ and DZ twin pairs were not significantly different for age and body mass index (BMI), and all of them were females. The number of twin pairs used in this study is sufficient to detect with 95% power heritability of 0.4 or higher with *P*<0.05 for a range of shared environmental contributions from low (0.1) to high (0.5) [15].

**Table 1:** Demographic characteristics of the study population, *mean (SD*).

**Phenotype**	**MZ**	**DZ**	**P**
N	440	610	
age, yrs	58.71(9.37)	57.83(9.61)	0.14
BMI, kg/m^2^	26.65(4.85)	26.50(4.68)	0.60

In total 76 glycan traits were studied which were derived from 24 directly measured glycan structures and derived measures. A detailed description of the glycan structures and the derived measures can be found in Lauc et al. [14]. For example, the Fc region of IgG contains two highly conserved asparagine (Asn-297) residues where complex core-fucosylated glycans such as FA2 (GP4) , FA2G1 (GP8, GP9) and FA2G2 (GP14) are found [14]. These carbohydrates are critical components of the Fc-Fcγ receptor interaction [1]. 

The heritability analyses for the 76 IgG patterns are presented in Table **2**. The best fitting model for the majority of the IgG pattern was the AE model (including additive genetic and non-shared environment), ascribing the total variance to additive genetic factors and non-shared environmental factors, with heritability estimates ranging from 0.49 for GP21 (which reflects the proportion of the A2G2S2 glycan, i.e. disialylated, digalactosylated, bi-antennary N-linked glycans; see Table **S1** for description of glycan codes) to 0.80 for GP8n (which reflects the proportion of the monogalactosylated isomer FA2[6]G1 relative to all the neutral glycans). The ACE model (including additive genetic, common environment and unique environments) was the best fitting model for 18 glycan traits with heritability estimates ranging from 0.19 for FtotalS1/FtotalS2 to 0.52 for FBG0n/G0n. In contrast, 7 glycan traits showed no clear genetic influence and appeared to be affected primarily by common and unique environmental factors. We have classified these 7, added to the 5 glycan traits in Table **2** with an additive component under 0.35, as “low heritability”.

**Table 2:** Heritability estimates and 95% confidence intervals for IgG glycan traits adjusted for age and batch.

	**MZ**		**DZ**					
**Glycan Trait**	Mean(SD)*	**ICC[95%CI]**	Mean(SD)*	**ICC[95%CI]**	**Best model**	**A[95%CI]**	**C[95%CI]**	**E[95%CI]**
GP1	0.1(0.06)	0.54[0.44,0.63]	0.11(0.06)	0.38[0.28,0.47]	AE	0.58[0.49,0.65]		0.42[0.35,0.51]
GP2	0.48(0.27)	0.68[0.61,0.75]	0.53(0.34)	0.24[0.13,0.34]	AE	0.72[0.64,0.48]		0.28[0.22,0.36]
GP4	19.41(6.45)	0.73[0.66,0.79]	19.26(5.63)	0.39[0.29,0.49]	AE	0.70[0.64,0.75]		0.30[0.25,0.36]
GP5	0.32(0.15)	0.63[0.54,0.71]	0.4(0.17)	0.41[0.32,0.50]	AE	0.72[0.65,0.77]		0.28[0.23,0.35]
GP6	5.33(1.77)	0.76[0.70,0.82]	5.32(1.68)	0.33[0.23,0.43]	AE	0.75[0.69,0.79]		0.25[0.21,0.31]
GP7	0.54(0.24)	0.66[0.58,0.73]	0.62(0.31)	0.33[0.23,0.43]	AE	0.73[0.67,0.79]		0.27[0.21,0.33]
GP8	18.97(1.74)	0.73[0.67,0.80]	19.19(1.83)	0.30[0.20,0.41]	AE	0.74[0.68,0.79]		0.26[0.21,0.32]
GP9	9.98(1.42)	0.74[0.68,0.80]	9.99(1.44)	0.41[0.32,0.51]	AE	0.75[0.69,0.79]		0.25[0.21,0.31]
GP10	6(1.21)	0.76[0.71,0.82]	5.99(1.19)	0.37[0.28,0.47]	AE	0.76[0.70,0.80]		0.24[0.20,0.30]
GP11	0.84(0.22)	0.73[0.67,0.79]	0.86(0.2)	0.38[0.28,0.48]	AE	0.71[0.65,0.76]		0.28[0.24,0.35]
GP12	0.67(0.32)	0.54[0.44,0.63]	0.71(0.38)	0.32[0.22,0.43]	AE	0.60[0.51,0.67]		0.40[0.33,0.49]
GP13	0.5(0.22)	0.64[0.57,0.72]	0.62(0.23)	0.46[0.37,0.55]	AE	0.70[0.63,0.75]		0.30[0.25,0.37]
GP14	14.33(4.96)	0.78[0.72,0.83]	13.6(4.22)	0.50[0.42,0.58]	ACE	0.36[0.19,0.55]	0.37[0.19,0.52]	0.27[0.22,0.33]
GP15	1.86(0.69)	0.75[0.69,0.81]	1.79(0.56)	0.55[0.47,0.63]	CE		0.66[0.61,0.70]	0.34[0.30,0.39]
GP16	3.3(0.74)	0.78[0.73,0.83]	3.45(0.65)	0.48[0.39,0.56]	ACE	0.45[0.28,0.65]	0.29[0.10,0.45]	0.26[0.21,0.32]
GP17	0.94(0.26)	0.60[0.51,0.68]	0.98(0.28)	0.32[0.22,0.42]	AE	0.62[0.54,0.68]		0.38[0.32,0.46]
GP18	9.54(3.28)	0.77[0.71,0.82]	9.68(2.86)	0.39[0.30,0.49]	AE	0.73[0.68,0.78]		0.27[0.22,0.32]
GP19	1.86(0.42)	0.54[0.45,0.64]	1.89(0.44)	0.44[0.35,0.53]	ACE	0.27[0.03,0.50]	0.29[0.10,0.47]	0.44[0.36,0.53]
GP21	1.32(0.41)	0.48[0.36,0.60]	1.31(0.42)	0.28[0.06,0.50]	AE	0.49[0.37,0.60]		0.51[0.40,0.63]
GP22	0.15(0.08)	0.27[0.14,0.39]	0.17(0.08)	0.28[0.18,0.38]	CE		0.28[0.20,0.36]	0.72[0.64,0.80]
GP23	1.65(0.53)	0.61[0.53,0.69]	1.65(0.54)	0.44[0.35,0.53]	ACE	0.37[0.14,0.59]	0.25[0.05,0.43]	0.38[0.32,0.47]
GP24	1.93(0.57)	0.53[0.43,0.62]	1.93(0.6)	0.51[0.42,0.59]	CE		0.51[0.45,0.57]	0.49[0.43,0.55]
FGS/(FG+FGS)	24.89(4.6)	0.76[0.70,0.81]	25.51(3.77)	0.41[0.31,0.50]	ACE	0.49[0.28,0.71]	0.21[0.01,0.39]	0.30[0.25,0.36]
FBGS/(FBG+FBGS)	30.66(6.62)	0.64[0.56,0.72]	30.91(6.89)	0.51[0.42,0.59]	ACE	0.32[0.12,0.52]	0.34[0.16,0.49]	0.34[0.28,0.42]
FGS/(F+FG+FGS)	18.68(4.63)	0.72[0.66,0.77]	19.2(4.07)	0.36[0.27,0.46]	AE	0.69[0.63,0.74]		0.31[0.26,0.37]
FBGS/(FB+FBG+FBGS)	21.64(5.2)	0.63[0.55,0.71]	21.86(5.57)	0.49[0.41,0.58]	ACE	0.35[0.15,0.56]	0.30[0.12,0.46]	0.35[0.28,0.42]
FG1S1/(FG1+FG1S1)	10.24(2.2)	0.76[0.70,0.81]	10.57(1.86)	0.45[0.36,0.54]	ACE	0.42[0.24,0.63]	0.29[0.09,0.45]	0.29[0.24,0.35]
FG2S1/(FG2+FG2S1+FG2S2)	37.65(6.68)	0.84[0.80,0.88]	38.9(4.76)	0.67[0.61,0.73]	CE		0.77[0.73,0.80]	0.23[0.20,0.27]
FG2S2/(FG2+FG2S1+FG2S2)	6.69(1.97)	0.64[0.57,0.72]	6.82(2.15)	0.44[0.35,0.53]	AE	0.69[0.63,0.75]		0.31[0.25,0.37]
FBG2S1/(FBG2+FBG2S1+FBG2S2)	33.2(5.86)	0.68[0.61,0.75]	33.84(4.86)	0.52[0.44,0.60]	CE		0.61[0.55,0.66]	0.39[0.34,0.45]
FBG2S2/(FBG2+FBG2S1+FBG2S2)	33.97(6.12)	0.49[0.39,0.59]	34.12(5.78)	0.48[0.39,0.57]	CE		0.48[0.41,0.54]	0.52[0.46,0.59]
FtotalS1/FtotalS2	4.34(1.36)	0.70[0.63,0.77]	4.44(1.26)	0.55[0.47,0.63]	ACE	0.19[0.01,0.37]	0.48[0.32[0.62]	0.33[0.27,0.40]
FS1/FS2	8.29(2.59)	0.74[0.68,0.80]	8.52(2.54)	0.53[0.45,0.61]	ACE	0.39[0.22,0.57]	0.34[0.18,0.49]	0.27[0.22,0.33]
FBS1/FBS2	1.01(0.25)	0.44[0.33,0.55]	1.02(0.21)	0.44[0.35,0.53]	CE		0.44[0.36,0.51]	0.56[0.49,0.64]
FBStotal/FStotal	0.28(0.1)	0.66[0.59,0.74]	0.27(0.09)	0.43[0.34,0.52]	ACE	0.23[0.02,0.46]	0.37[0.17,0.55]	0.40[0.17,0.55]
FBS1/FS1	0.16(0.06)	0.67[0.59,0.74]	0.15(0.05)	0.43[0.34,0.52]	ACE	0.39[0.18,0.61]	0.26[0.06,0.43]	0.35[0.29.0.43]
FBS1/(FS1+FBS1)	0.13(0.04)	0.69[0.62,0.76]	0.13(0.04)	0.43[0.34,0.53]	ACE	0.42[0.22,0.64]	0.25[0.05,0.41]	0.33[0.27,0.40]
FBS2/FS2	1.22(0.32)	0.67[0.60,0.74]	1.21(0.28)	0.20[0.09,0.31]	AE	0.61[0.54,0.68]		0.39[0.32,0.46]
FBS2/(FS2+FBS2)	0.54(0.06)	0.66[0.58,0.73]	0.54(0.06)	0.23[0.12,0.34]	AE	0.61[0.54,0.68]		0.39[0.32,0.46]
GP1n	0.12(0.07)	0.52[0.41,0.62]	0.13(0.08)	0.37[0.27,0.47]	AE	0.57[0.48,0.65]		0.43[0.35,0.52]
GP2n	0.6(0.34)	0.68[0.61,0.75]	0.67(0.41)	0.24[0.13,0.34]	AE	0.71[0.64,0.77]		0.29[0.23,0.36]
GP4n	24.29(7.41)	0.74[0.68,0.80]	24.21(6.4)	0.42[0.32,0.51]	ACE	0.49[0.29,0.70]	0.21[0.01,0.38]	0.30[0.25,0.36]
GP5n	0.41(0.18)	0.59[0.51,0.68]	0.51(0.21)	0.40[0.31,0.49]	AE	0.69[0.62,0.75]		0.31[0.25,0.38]
GP6n	6.65(2)	0.76[0.71,0.82]	6.67(1.9)	0.34[0.24,0.44]	AE	0.75[0.70,0.80]		0.25[0.20,0.30]
GP7n	0.68(0.3)	0.65[0.58,0.73]	0.78(0.39)	0.32[0.22,0.42]	AE	0.73[0.66,0.78]		0.27[0.22,0.34]
GP8n	23.96(2.42)	0.80[0.75,0.85]	24.33(2.45)	0.36[0.27,0.46]	AE	0.80[0.76,0.84]		0.20[0.16,0.24]
GP9n	12.59(1.72)	0.76[0.70,0.81]	12.66(1.77)	0.42[0.33,0.51]	AE	0.76[0.71,0.81]		0.24[0.19,0.29]
GP10n	7.55(1.4)	0.75[0.69,0.81]	7.58(1.44)	0.37[0.27,0.46]	AE	0.76[0.70,0.80]		0.24[0.20,0.30]
GP11n	1.06(0.24)	0.70[0.63,0.77]	1.09(0.23)	0.36[0.26,0.46]	AE	0.70[0.63,0.75]		0.30[0.25,0.37]
GP12n	0.85(0.42)	0.54[0.45,0.64]	0.92(0.53)	0.30[0.20,0.41]	AE	0.61[0.52,0.68]		0.39[0.32,0.48]
GP13n	0.63(0.29)	0.63[0.55,0.71]	0.79(0.31)	0.44[0.35,0.53]	AE	0.70[0.63,0.75]		0.30[0.25,0.37]
GP14n	18.23(6.63)	0.77[0.72,0.82]	17.39(5.83)	0.46[0.37,0.55]	ACE	0.47[0.29,0.67]	0.26[0.07,0.42]	0.27[0.22,0.33]
GP15n	2.36(0.86)	0.73[0.67,0.79]	2.28(0.74)	0.51[0.42,0.59]	ACE	0.24[0.06,0.43]	0.44[0.27,0.59]	0.32[0.26,0.38]
G0n	31.68(9.06)	0.75[0.69,0.81]	31.68(7.96)	0.40[0.30,0.49]	AE	0.72[0.66,0.76]		0.28[0.24,0.34]
G1n	45.84(2.78)	0.68[0.61,0.75]	46.44(2.79)	0.33[0.23,0.43]	AE	0.69[0.62,0.74]		0.31[0.26,0.38]
G2n	22.08(7.72)	0.76[0.70,0.81]	21.37(6.83)	0.48[0.39,0.56]	ACE	0.41[0.23,0.61]	0.31[0.12,0.46]	0.28[0.23,0.34]
Fn total	96.81(1.06)	0.55[0.46,0.64]	96.33(1.36)	0.32[0.21,0.42]	AE	0.64[0.55,0.71]		0.36[0.29,0.45]
FG0n total/G0n	98.07(0.97)	0.57[0.48,0.66]	97.89(1.08)	0.27[0.16,0.37]	AE	0.60[0.51,0.67]		0.40[0.33,0.49]
FG1n total/G1n	98.52(0.65)	0.64[0.56,0.72]	98.31(0.86)	0.33[0.23,0.43]	AE	0.72[0.65,0.78]		0.28[0.22,0.35]
FG2n total /G2n	93.04(2.25)	0.52[0.43,0.62]	91.87(2.69)	0.33[0.23,0.43]	AE	0.63[0.54,0.70]		0.37[0.30,0.46]
Fn	79.19(3.33)	0.70[0.63,0.77]	78.71(3.53)	0.36[0.26,0.46]	AE	0.72[0.66,0.77]		0.28[0.23,0.34]
FG0n/G0n	76.69(4.51)	0.71[0.65,0.78]	76.62(4.25)	0.41[0.32,0.50]	AE	0.70[0.64,0.75]		0.30[0.25,0.36]
FG1n/G1n	79.72(3.44)	0.73[0.66,0.79]	79.62(3.59)	0.37[0.28,0.47]	AE	0.74[0.68,0.79]		0.26[0.21,0.32]
FG2n/G2n	82.16(3.32)	0.56[0.47,0.65]	81.03(3.59)	0.28[0.18,0.39]	AE	0.61[0.52,0.68]		0.39[0.32,0.48]
FBn	17.62(2.98)	0.76[0.71,0.82]	17.62(2.93)	0.37[0.28,0.47]	AE	0.76[0.71,0.81]		0.24[0.19,0.29]
FBG0n/G0n	21.38(4.24)	0.75[0.69,0.81]	21.26(3.78)	0.43[0.34,0.52]	ACE	0.52[0.33,0.73]	0.20[0.01,0.37]	0.27[0.29,0.34]
FBG1n/G1n	18.8(3.29)	0.76[0.70,0.81]	18.69(3.3)	0.39[0.29,0.48]	AE	0.76[0.71,0.80]		0.24[0.20,0.29]
FBG2n/G2n	10.88(2.07)	0.70[0.63,0.77]	10.81(1.86)	0.34[0.25,0.44]	AE	0.68[0.61,0.73]		0.32[0.27,0.39]
FBn/Fn	0.22(0.05)	0.75[0.69,0.81]	0.23(0.05)	0.36[0.26,0.46]	AE	0.75[0.69,0.79]		0.25[0.21,0.31]
FBn/Fn total	18.21(3.12)	0.75[0.69,0.81]	18.3(3.12)	0.36[0.27,0.47]	AE	0.76[0.70,0.80]		0.24[0.20,0.30]s
Fn/(Bn + FBn)	4.49(0.94)	0.74[0.69,0.80]	4.42(0.93)	0.43[0.33,0.52]	AE	0.75[0.69,0.79]		0.25[0.21,0.31]
Bn/(Fn + FBn) ‰	6.54(3.06)	0.62[0.54,0.70]	8.19(3.34)	0.44[0.35,0.53]	AE	0.69[0.62,0.74]		0.31[0.26,0.38]
FBG2n/FG2n	0.13(0.03)	0.68[0.60,0.75]	0.13(0.03)	0.31[0.21,0.41]	AE	0.66[0.59,0.72]		0.34[0.28,0.41]
FBG2n /(FG2n + FBG2n )	11.71(2.3)	0.68[0.61,0.75]	11.79(2.13)	0.32[0.22,0.42]	AE	0.66[0.59,0.72]		0.34[0.28,0.41]
FG2n/(BG2n + FBG2n)	6.17(1.36)	0.70[0.64,0.77]	5.72(1.15)	0.38[0.29,0.48]	AE	0.69[0.62,0.74]		0.31[0.26,0.38]
BG2n/(FG2n + FBG2n) ‰	32.44(15.27)	0.62[0.54,0.70]	42.02(16.45)	0.43[0.34,0.52]	AE	0.73[0.67,0.78]		0.27[0.22,0.33]

Values in the three rightmost columns indicate the amount of variance attributed to the compartment of additive genetic factors (A or heritability), common/shared environmental factors (C) and unique environmental factors (E). ICC = intra-class correlation coefficient. 95% confidence intervals for both ICC and ACE are reported.

^*^ means and SD reported are unadjusted

51 of the 76 glycan traits (67%) studied have an additive genetic component of 0.5 or greater, meaning that at least half of the variance in their levels is determined by genetic factors, 5 glycan traits had a genetic contribution under 0.35. Heritability estimates for the remaining glycan traits varied between 0.35 and 0.5.

We hypothesized that IgG glycans showing none or low genetic influences could be epigenetically mediated and we compared their levels with genome-wide DNA methylation profiles from the Illumina HumanMethylation27 DNA Analysis BeadChip assay in 127 MZ and DZ female twins [16]. The analyses were adjusted for age, sex, BMI, methylation chip, sample position on methylation chip, and family relatedness. 

Among glycan traits with a low heritability we identified 2 CpG-sites (cg08392591, cg26991199) at which DNA methylation levels were associated with levels of 4 IgG glycan traits with *P* <2 x 10^-6^ (the Bonferroni cut off for array-wide significance; see Table **3**). Probe cg08392591 maps to a CpG island 5’ from the *ANKRD11* gene on chromosome 16 while cg26991199 maps to *SRSF10* gene on chromosome 1. *ANKRD11* is a p53 activator, while *SFRS10* is involved in constitutive and regulated RNA splicing and in particular is involved in regulation of splicing of mRNA precursors upon heat shock. 

**Table 3:** Association between IgG levels and methylation probes (*P*<2x10^-6^).

**Probe**	**Chr**	**Map position(hg 18 B36)**	**nearest gene**	**Glycan**	**Beta**	**SE**	***P***	**h^2^**	**h^2^>0.35**
cg13782134	16	67919362	*NRN1L*	FBS2/FS2	-0.36	0.05	1.19x10^-9^	0.61	yes
cg08392591	16	89556376	*ANKRD11*	FBGS/(FB+FBG+FBGS)	0.35	0.06	3.05x10^-8^	0.35	no
cg13782134	16	67919362	*NRN1L*	FBS2/(FS2+FBS2)	-0.33	0.05	5.8x10^-8^	0.61	yes
cg16029957	2	37425956	*QPCT*	GP13n	-0.19	0.03	2x10^-7^	0.70	yes
cg16029957	2	37425956	*QPCT*	Bn/(Fn + FBn) ‰	-0.19	0.03	3.57x10^-7^	0.69	yes
cg26991199	1	24307153	*SFRS10*	GP24	0.24	0.04	8.99x10^-7^	-	no
cg26991199	1	24307153	*SFRS10*	FBGS/(FB+FBG+FBGS)	0.28	0.05	1.01x10^-6^	0.35	no
cg16029957	2	37425956	*QPCT*	GP13	-0.17	0.03	1.44x10^-6^	0.70	yes
cg26991199	1	24307153	*SFRS10*	GP19	0.27	0.05	1.47x10^-6^	0.27	no
cg08392591	16	89556376	*ANKRD11*	FBGS/(FBG+FBGS)	0.34	0.07	1.67x10^-6^	0.32	no

Among the 64 glycan traits that had heritabilities above 0.35 we observed five array-wide significant hits (Table **3**) for the two probes, cg13782134 (mapping to the *NRN1L* gene) and cg16029957 (mapping near the *QPCT* gene). *NRN1L* encodes a neuritin-like protein precursor and its role in immunoglobulin glycosylation is unclear. *QPCT* encodes the human pituitary glutaminyl cyclase, which is responsible for the presence of pyroglutamyl residues in many neuroendocrine peptides.

Overall, the proportion of array-wide epigenetic associations was significantly larger (*P*<0.005) among glycan traits with low heritability (5/12= 41.6%) than in those showing high heritability (4/64 = 6.2%).

We also tested if the glycan traits measured could have some clinical relevance and we found that 4 of the 76 glycan traits are significantly (after adjustment for multiple tests) associated with circulating levels of triglycerides, a well known risk factor of cardiovascular risk [17] and 3 are associated with circulating levels of C-reactive protein (CRP), a well known marker of systemic inflammation [18] (Table **S2**). Although the investigation of the role of the various glycans in health and disease is beyond the scope of our study, these data suggest that the molecular mechanisms underlying IgG glycans can yield clinically relevant insights. We note that all the glycans associated with these two traits have high heritabilities and no epigenetic significant associations were found for these. However, we did not carry out a systematic study of the relationship between IgG glycans and cardiovascular or inflammatory traits which may show associations also among glycans with low heritabilities.

## Discussion

In this study we have evaluated, using a classical study design, the heritable and non-heritable component of circulating immunoglobulin G glycome composition. The study was sufficiently powered to detect with 95% probability heritabilities above 0.4 even with low-shared environmental contributions and we detected 59 glycan traits with heritabilities above this cut-off.

Our data show that variation in levels of 51 of the 76 IgG glycan traits studied is at least half heritable and only a small proportion of N-glycan traits have a low genetic contribution. Though glycans are produced in a complex biosynthetic pathway [19] and were believed to be significantly affected by many environmental factors [20], we find a high contribution of the genetic component to IgG glycome composition. Compared to GWAS study of the plasma glycome in 2705 individuals [13], the recent GWAS study of the IgG glycome in 2247 [14] has identified five times more genetic loci with genome-wide significant associations. Inter-individual variation of the IgG glycome [9] is more than three-fold larger than the inter-individual variation of the plasma glycome [10]. Large heritability and the large number of involved genetic loci suggest that the genetic regulation of the IgG glycome is stronger than the regulation of the total plasma glycome. Fine details of IgG glycan structure significantly affect function of immunoglobulins [21], thus close regulation of IgG glycosylation is required for proper function of the immune system [22]. Genetic loci with variants that affect IgG glycome composition were reported for the majority of glycans with high heritability [12-14] (Table **S3**), and some of the highly heritable glycans seem to be affected by multiple loci (e.g., FG0n/G0n associates with genetic variants in the following genes: *FUT8*, *MGAT3*, *IKZF1* and *SMARCB1*-*DERL3*).

In addition to the genetic contribution to the glycome, an important source of complexity and variability in IgG glycosylation is the interaction with the environment, some of which may be revealed by epigenetic changes [23]. Epigenetic silencing of *HNF1A*, a known master regulator of plasma protein fucosylation, has been shown to be associated with changes in the composition of the human plasma N-glycome [24]. In this study we find that methylation levels at other genes are also implicated in glycome composition, both in those with high heritabilities and those with a lower genetic contribution. By using a well-characterized cohort with epigenetics data available, such as TwinsUK, it was possible to integrate glycome data with other existing molecular data and adjust for confounders. 

The main limitation of the present study is that due to the novelty of the glycan phenotypes we lack the replication for the epigenetic findings in an independent cohort. The fact that we find epigenome-wide significant hits on a relatively small sample suggests that epigenetic factors contribute to IgG glycan levels, although due to the lack of replication we cannot exclude false positive results. Epigenetic factors also play a role in the case of some glycans with a high heritability. However, we only found 5 significant methylation hits (mapping to two probes) for 64 glycan traits with h^2^ >0.35 and 5 significant hits (mapping to two probes) for 12 glycan traits with h^2^≤0.35. The probes associated with highly heritable glycan traits were different to those associated with glycan traits with lower heritabilities. 

For glycan traits with lower heritabilities the most significant probe maps to the p53 activator *ANKRD11*. The tumor suppressor p53 is known to be able to modulate innate immune gene responses. For glycan traits with high heritabilities the two hits appear related to neuroendocrine regulation, in one case directly to a glycosylation enzyme *QPCT*, in the second case to a neuritin-like protein precursor which has been implicated in neuronal survival [25]. These genes have not been previously reported to have a role in IgG glycosylation. A similar observation was recently reported in a GWA study of the IgG glycome which identified 12 genes not previously known to be involved in IgG glycosylation [14], providing further evidence that IgG glycosylation is a very complex and tightly regulated process. As more epigenetic and genetic data become available for cohorts with IgG *N-*glycan characterizations it will become possible to elucidate the molecular pathways underlying many complex traits.

## Materials and Methods

### Ethic statement

The study was approved by St. Thomas’ Hospital Research Ethics Committee, and all twins provided informed written consent.

### Study subjects

Study subjects were twins enrolled in the TwinsUK registry, a national register of adult twins. Twins were recruited as volunteers by successive media campaigns without selecting for particular diseases or traits [26]. All twin pairs recruited were of the same sex. 

In this study we analysed data from 440 monozygotic and 610 dizygotic female twins with glycomics and epigenomic data available.

### Isolation of IgG from human plasma

The IgG was isolated using protein G monolithic plates as described previously [9]. Briefly, 90 µl of plasma was diluted 10x with PBS, applied to the protein G plate (BIA Separations, Ljubljana, Slovenia) and instantly washed. IgGs were eluted with 1 ml of 0.1 M formic acid and neutralized with 1 M ammonium bicarbonate.

### *N*-Glycan Release

Isolated IgG samples were dried in a vacuum centrifuge. After drying, proteins were denatured with addition of 20 μL 2% SDS (w/v) (Invitrogen, Carlsbad, CA, USA) and by incubation at 60 °C for 10 min. Subsequently, 10 μL of 4% Igepal-CA630 (Sigma-Aldrich, St. Louis, MO, USA) and 0.5 mU of PNGase F in 10 μL 5× PBS were added to the samples. The samples were incubated overnight at 37 °C for *N*-glycan release.

### 2-aminobenzamide labelling

The released *N*-glycans were labelled with 2-aminobenzamide (2-AB), the fluorescent dye used to make glycans visible in UPLC. The labelling mixture was freshly prepared by dissolving 2-AB (Sigma-Aldrich, St. Louis, MO, USA) in DMSO (Sigma-Aldrich, St. Louis, MO, USA) and glacial acetic acid (Merck, Darmstadt, Germany) mixture (85:15, v/v) to a final concentration of 48 mg/mL. A volume of 25 μL of labelling mixture was added to each *N*-glycan sample in the 96-well plate. Also, 25 μL of freshly prepared reducing agent solution (2-picoline borane (Sigma-Aldrich, St. Louis, MO, USA) in DMSO – concentration of 106.96 mg/ml) was added and the plate was sealed using adhesive tape. Mixing was achieved by shaking for 10 min, followed by 2 hour incubation at 65 °C. Samples (in a volume of 100 μL) were brought to 80% ACN (v/v) by adding 400 μL of ACN.

### Cleaning and elution of labelled glycans using HILIC-Solid Phase Extraction (SPE)

Free label and reducing agent were removed from the samples using HILIC-SPE. An amount of 200 μL of 0.1 g/mL suspension of microcrystalline cellulose (Merck, Darmstadt, Germany) in water was applied to each well of a 0.45 μm GHP filter plate (Pall Corporation, Ann Arbor, MI, USA). Solvent was removed by application of vacuum using a vacuum manifold (Millipore Corporation, Billerica, MA, USA). All wells were prewashed using 5 × 200 μL water, followed by equilibration using 3 × 200 μL acetonitrile/water (80:20, v/v). The samples were loaded to the wells. The wells were subsequently washed 5 × using 200 μL acetonitrile/water (80:20, v/v).

Glycans were eluted 2 × with 100 μL of water and combined elutes were not dried, but either analyzed immediately by UPLC or stored at –20 °C until usage.

### Hydrophilic interaction high performance liquid chromatography (HILIC)

Fluorescently labelled *N*-glycans were separated by hydrophilic interaction chromatography on a Waters Acquity UPLC instrument (Milford, MA, USA) consisting of a quaternary solvent manager, sample manager and a FLR fluorescence detector set with excitation and emission wavelengths of 330 and 420 nm, respectively. The instrument was under the control of Empower 2 software, build 2145 (Waters, Milford, MA, USA). Labeled *N*-glycans (10 μl) were separated on a Waters BEH Glycan chromatography column, 100 × 2.1 mm i.d., 1.7 μm BEH particles, with 100 mM ammonium formate, pH 4.4, as solvent A and acetonitrile as solvent B. Separation method used linear gradient of 75–62% acetonitrile (v/v) at flow rate of 0.4 ml/min in a 25 min analytical run. Samples were maintained at 5 °C before injection, and the separation temperature was 60 °C. The system was calibrated using an external standard of hydrolyzed and 2-AB labelled glucose oligomers from which the retention times for the individual glycans were converted to glucose units. Data processing was performed using an automatic processing method with a traditional integration algorithm after which each chromatogram was manually corrected to maintain the same intervals of integration for all the samples. The chromatograms obtained were all separated in the same manner into 24 peaks and the amount of glycans in each peak was expressed as % of total integrated area. In addition to 24 directly measured glycan structures, 53 derived traits were calculated as described previously [9] (see Table **S1**). These derived traits average particular glycosylation features (galactosylation, fucosylation, sialylation) across different individual glycan structures. Consequently, they are more closely related to individual enzymatic activities, and underlying genetic polymorphisms [13].

### Epigenetics

DNA methylation levels were obtained using the 27k Illumina CpG methylation probe array in 127 female twins aged 32 to 80 randomly selected from the discovery cohort. QC measure were applied, as previously described [16] and 24,641 autosomal probes passed quality control and were included in the analysis. Probes were standardized to have mean zero and variance 1.

### Statistical methods

Statistical analysis was carried out using Stata version 11. The R package OpenMX was used to calculate heritability. 

Heritability of glycome composition was estimated using structural equation modelling to decompose the observed phenotypic variance into three latent sources of variation: additive genetic variance (A), shared/common environmental variance (C), and non-shared/unique environmental variance (E) [27] adjusting for age and batch. Additive genetic influences are indicated when monozygotic twins are more similar than dizygotic twins. The common environmental component estimates the contribution of family environment which is assumed to be equal in both MZ and DZ twin pairs [28], whereas the unique environmental component does not contribute to twin similarity, rather it estimates the effects that apply only to each individual and includes measurement error. The equal environment assumption across zygosities implies that any greater similarity between MZ twins than DZ twins is attributed to greater sharing of genetic influences.

The best fitting model (among ACE, AE, CE, and E models) was determined by removing each factor sequentially from the full model and testing the deterioration in fit of the various nested models, using the likelihood ratio test (p=0.05). In addition, the Akaike Information Criteria (AIC) was considered, with lower values indicating the most suitable model. The AIC combines the goodness of fit of a model (the discrepancy of expected to observed covariance matrixes) with its simplicity, resulting in a measure of parsimony [27]. The most parsimonious model was then used to estimate heritability, defined as the proportion of the phenotypic variation attributable to genetic factors

Random intercept logistic regression was used to test the association between whole-blood DNA methylation patterns and IgG patterns with low heritability, adjusting for age, sex, BMI, methylation chip, sample position on methylation chip, and family relatedness. Adjusting for zygosity did not change the results.

## Supporting Information

Table S1 **Description of the glycan codes.**
(DOCX)

Table S2 **List of glycans associated with circulating levels of triglycerides (log) and of C-reactive protein (Bonferroni *P*<7x10^-4^).**
(DOCX)

Table S3 **List of loci associated with all glycans with high heritability (h^2^>0.55).**
(DOCX)
