# Supplementary material for: Glycosylation of Immunoglobulin G: Role of Genetic and Epigenetic Influences
Source: PLoS One. 2013 Dec 6;8(12):e82558. doi: 10.1371/journal.pone.0082558 (PMC3855797; doi:10.1371/journal.pone.0082558)
Supplement: Table S1 — Description of the glycan codes. (DOCX) [file pone.0082558.s001.docx]

**Table S1. Description of the glycan codes (This Table is derived from Table S1, Lauc et al, Plos Genetics 2013, [14]).**

| **Glycan Code** | **DESCRIPTION** | **FORMULA** |
| --- | --- | --- |
| GP1 | *The percentage of FA1 glycan in total IgG glycans* | *GP1 / GP× 100* |
| GP2 | *The percentage of A2 glycan in total IgG glycans* | *GP2 / GP× 100* |
| GP4 | *The percentage of FA2 glycan in total IgG glycans* | *GP4 / GP× 100* |
| GP5 | *The percentage of M5 glycan in total IgG glycans* | *GP5 / GP× 100* |
| GP6 | *The percentage of FA2B glycan in total IgG glycans* | *GP6 / GP× 100* |
| GP7 | *The percentage of A2G1 glycan in total IgG glycans* | *GP7 / GP× 100* |
| GP8 | *The percentage of FA2[6]G1 glycan in total IgG glycans* | *GP8 / GP× 100* |
| GP9 | *The percentage of FA2[3]G1 glycan in total IgG glycans* | *GP9 / GP× 100* |
| GP10 | *The percentage of FA2[6]BG1 glycan in total IgG glycans* | *GP10 / GP× 100* |
| GP11 | *The percentage of FA2[3]BG1 glycan in total IgG glycans* | *GP11 / GP× 100* |
| GP12 | *The percentage of A2G2 glycan in total IgG glycans* | *GP12 / GP× 100* |
| GP13 | *The percentage of A2BG2 glycan in total IgG glycans* | *GP13 / GP× 100* |
| GP14 | *The percentage of FA2G2 glycan in total IgG glycans* | *GP14 / GP× 100* |
| GP15 | *The percentage of FA2BG2 glycan in total IgG glycans* | *GP15 / GP× 100* |
| GP16 | *The percentage of FA2G1S1 glycan in total IgG glycans* | *GP16 / GP × 100* |
| GP17 | *The percentage of A2G2S1 glycan in total IgG glycans* | *GP17/ GP × 100* |
| GP18 | *The percentage of FA2G2S1 glycan in total IgG glycans* | *GP18 / GP × 100* |
| GP19 | *The percentage of FA2BG2S1 glycan in total IgG glycans* | *GP19 / GP × 100* |
| GP20 | *Structure not determined* | *GP20 / GP × 100* |
| GP21 | *The percentage of A2G2S2 glycan in total IgG glycans* | *GP21 / GP × 100* |
| GP22 | *The percentage of A2BG2S2 glycan in total IgG glycans* | *GP22 / GP × 100* |
| GP23 | *The percentage of FA2G2S2 glycan in total IgG glycans* | *GP23 / GP × 100* |
| GP24 | *The percentage of FA2BG2S2 glycan in total IgG glycans* | *GP24 / GP × 100* |
| FGS/(FG+FGS) | *The percentage of sialylation of fucosylated galactosylated structures without bisecting GlcNAc in total IgG glycans* | *Ʃ(GP16 + GP18 + GP23) / Ʃ(GP16 + GP18 + GP23 + GP8 + GP9 + GP14)× 100* |
| FBGS/(FBG+FBGS) | *The percentage of sialylation of fucosylated galactosylated structures with bisecting GlcNAc in total IgG glycans* | *Ʃ(GP19 + GP24) / Ʃ(GP19 + GP24 + GP10 + GP11 + GP15)× 100* |
| FGS/(F+FG+FGS) | *The percentage of sialylation of all fucosylated structures without bisecting GlcNAc in total IgG glycans* | *Ʃ(GP16 + GP18 + GP23) / Ʃ(GP16 + GP18 + GP23 + GP4 + GP8 + GP9 + GP14)× 100* |
| FBGS/(FB+FBG+FBGS) | *The percentage of sialylation of all fucosylated structures with bisecting GlcNAc in total IgG glycans* | *Ʃ(GP19 + GP24) / Ʃ(GP19 + GP24 + GP6 + GP10 + GP11 + GP15)× 100* |
| FG1S1/(FG1+FG1S1) | *The percentage of monosialylation of fucosylated monogalactosylated structures in total IgG glycans* | *GP16 / Ʃ(GP16 + GP8 + GP9)× 100* |
| FG2S1/(FG2+FG2S1+FG2S2) | *The percentage of monosialylation of fucosylated digalactosylated structures in total IgG glycans* | *GP18 / Ʃ(GP18 + GP14 + GP23)× 100* |
| FG2S2/(FG2+FG2S1+FG2S2) | *The percentage of disialylation of fucosylated digalactosylated structures in total IgG glycans* | *GP23 / Ʃ(GP23 + GP14 + GP18)× 100* |
| FBG2S1/(FBG2+FBG2S1+FBG2S2) | *The percentage of monosialylation of fucosylated digalactosylated structures with bisecting GlcNAc in total IgG glycans* | *GP19 / Ʃ(GP19 + GP15 + GP24)× 100* |
| FBG2S2/(FBG2+FBG2S1+FBG2S2) | *The percentage of disialylation of fucosylated digalactosylated structures with bisecting GlcNAc in total IgG glycans* | *GP24 / Ʃ(GP24 + GP15 + GP19)× 100* |
| F^total^S1/F^total^S2 | *Ratio of all fucosylated (+/- bisecting GlyNAc) monosialylated and disialylated structures in total IgG glycans* | *Ʃ(GP16 + GP18 + GP19) / Ʃ(GP23 + GP24)* |
| FS1/FS2 | *Ratio of fucosylated (without bisecting GlcNAc) monosialylated and disialylated structures in total IgG glycans* | *Ʃ(GP16 + GP18) / GP23* |
| FBS1/FBS2 | *Ratio of fucosylated (with bisecting GlcNAc) monosialylated and disialylated structures in total IgG glycans* | *GP19 / GP24* |
| FBS^total^/FS^total^ | *Ratio of all fucosylated sialylated structures with and without bisecting GlcNAc* | *Ʃ(GP19 + GP24) / Ʃ(GP16 + GP18 + GP23)* |
| FBS1/FS1 | *Ratio of fucosylated monosialylated structures with and without bisecting GlcNAc* | *GP19 / Ʃ(GP16 + GP18)* |
| FBS1/(FS1+FBS1) | *The incidence of bisecting GlcNAc in all fucosylated monosialylated structures in total IgG glycans* | *GP19 / Ʃ(GP16 + GP18 + GP19)* |
| FBS2/FS2 | *Ratio of fucosylated disialylated structures with and without bisecting GlcNAc* | *GP24 / GP23* |
| FBS2/(FS2+FBS2) | *The incidence of bisecting GlcNAc in all fucosylated disialylated structures in total IgG glycans* | *GP24 / Ʃ(GP23 + GP24)* |
|  |  | ***GP = Ʃ(GP1:GP24)*** |
| GP1^n^ | *The percentage of FA1 glycan in total neutral IgG glycans (GP^n^)* | *GP1 / GP^n^× 100* |
| GP2^n^ | *The percentage of A2 glycan in total neutral IgG glycans (GP^n^)* | *GP2 / GP^n^× 100* |
| GP4^n^ | *The percentage of FA2 glycan in total neutral IgG glycans (GP^n^)* | *GP4 / GP^n^× 100* |
| GP5^n^ | *The percentage of M5 glycan in total neutral IgG glycans (GP^n^)* | *GP5 / GP^n^× 100* |
| GP6^n^ | *The percentage of FA2B glycan in total neutral IgG glycans (GP^n^)* | *GP6 / GP^n^× 100* |
| GP7^n^ | *The percentage of A2G1 glycan in total Ineutral IgG glycans (GP^n^)* | *GP7 / GP^n^× 100* |
| GP8^n^ | *The percentage of FA2[6]G1 glycan in total neutral IgG glycans (GP^n^)* | *GP8 / GP^n^× 100* |
| GP9^n^ | *The percentage of FA2[3]G1 glycan in total neutral IgG glycans (GP^n^)* | *GP9 / GP^n^× 100* |
| GP10^n^ | *The percentage of FA2[6]BG1 glycan in total neutral IgG glycans (GP^n^)* | *GP10 / GP^n^× 100* |
| GP11^n^ | *The percentage of FA2[3]BG1 glycan in total neutral IgG glycans (GP^n^)* | *GP11 / GP^n^× 100* |
| GP12^n^ | *The percentage of A2G2 glycan in total neutral IgG glycans (GP^n^)* | *GP12 / GP^n^× 100* |
| GP13^n^ | *The percentage of A2BG2 glycan in total neutral IgG glycans (GP^n^)* | *GP13 / GP^n^× 100* |
| GP14^n^ | *The percentage of FA2G2 glycan in total neutral IgG glycans (GP^n^)* | *GP14 / GP^n^× 100* |
| GP15^n^ | *The percentage of FA2BG2 glycan in total neutral IgG glycans (GP^n^)* | *GP15 / GP^n^× 100* |
| G0^n^ | *The percentage of agalactosylated structures in total neutral IgG glycans* | *Ʃ(GP1^n^: GP6^n^)* |
| G1^n^ | *The percentage of monogalactosylated structures in total neutral IgG glycans* | *Ʃ(GP7^n^: GP11^n^)* |
| G2^n^ | *The percentage of digalactosylated structures in total neutral IgG glycans* | *Ʃ(GP12^n^: GP15^n^)* |
| F^n total^ | *The percentage of all fucosylated (+/- bisecting GlcNAc) structures in total neutral IgG glycans* | *Ʃ(GP1^n^+ GP4^n^+ GP5^n^+ GP6^n^+ GP8^n^+ GP9^n^+ GP10^n^+ GP11^n^+ GP14^n^+ GP15^n^)* |
| FG0^n total^/G0^n^ | *The percentage of fucosylation of agalactosylated structures* | *Ʃ(GP1^n^+ GP4^n^+ GP5^n^+ GP6^n^) / G0^n^ × 100* |
| FG1^n total^/G1^n^ | *The percentage of fucosylation of monogalactosylated structures* | *Ʃ(GP8^n^+ GP9^n^+ GP10^n^+ GP11^n^) / G1^n^ × 100* |
| FG2^n total^ /G2^n^ | *The percentage of fucosylation of digalactosylated structures* | *Ʃ(GP14^n^+ GP15) / G2^n^ × 100* |
| F^n^ | *The percentage of fucosylated (without bisecting GlcNAc) structures in total neutral IgG glycans* | *Ʃ(GP1^n^+ GP4^n^+ GP5^n^+ GP8^n^+ GP9^n^+ GP14^n^)* |
| FG0^n^/G0^n^ | *The percentage of fucosylation (without bisecting GlcNAc) of agalactosylated structures* | *Ʃ(GP1^n^+ GP4^n^+ GP5^n^) / G0^n^ × 100* |
| FG1^n^/G1^n^ | *The percentage of fucosylation (without bisecting GlcNAc) of monogalactosylated structures* | *Ʃ(GP8^n^+ GP9^n^) / G1^n^ × 100* |
| FG2^n^/G2^n^ | *The percentage of fucosylation (without bisecting GlcNAc) of digalactosylated structures* | *GP14^n^/ G2^n^ × 100* |
| FB^n^ | *The percentage of fucosylated (with bisecting GlcNAc) structures in total neutral IgG glycans* | *Ʃ(GP6^n^ + GP10^n^ + GP11^n^ + GP15^n^)* |
| FBG0^n^/G0^n^ | *The percentage of fucosylation (with bisecting GlcNAc) of agalactosylated structures* | *GP6^n^/ G0^n^ × 100* |
| FBG1^n^/G1^n^ | *The percentage of fucosylation (with bisecting GlcNAc) of monogalactosylated structures* | *Ʃ(GP10^n^ + GP11^n^) / G1^n^ × 100* |
| FBG2^n^/G2^n^ | *The percentage of fucosylation (with bisecting GlcNAc) of digalactosylated structures* | *GP15) / G2^n^ × 100* |
| FB^n^/F^n^ | *Ratio of fucosylated structures with and without bisecting GlcNAc* | *FB^n^/ F^n^ × 100* |
| FB^n^/F^n total^ | *The incidence of bisecting GlcNAc in all fucosylated structures in total neutral IgG glycans* | *FB^n^/ F^n^ ^total^ × 100* |
| F^n^/(B^n^ + FB^n^) | *Ratio of fucosylated non-bisecting GlcNAc structures and all structures with bisecting GlcNAc* | *F^n^/(GP13^n^ + FB^n^ )* |
| B^n^/(F^n^ + FB^n^) | *Ratio of structures with bisecting GlcNAc and all fucosylated structures (+/- bisecting GlcNAc)* | *GP13^n^/ (F^n^+ FB^n^ ) × 1000* |
| FBG2^n^/FG2^n^ | *Ratio of fucosylated digalactosylated structures with and without bisecting GlcNAc* | *GP15^n^/GP14^n^* |
| FBG2^n^ /(FG2^n^ + FBG2^n^ ) | *The incidence of bisecting GlcNAc in all fucosylated digalactosylated structures in total neutral IgG glycans* | *GP15^n^/(GP14^n^ + GP15^n^) × 100* |
| FG2^n^/(BG2^n^ + FBG2^n^) | *Ratio of fucosylated digalactosylated non-bisecting GlcNAc structures and all digalactosylated structures with bisecting GlcNAc* | *GP14^n^/(GP13^n^ + GP15^n^)* |
| BG2^n^/(FG2^n^ + FBG2^n^) | *Ratio of digalactosylated structures with bisecting GlcNAc and all fucosylated digalactosylated structures (+/- bisecting GlcNAc)* | *GP15^n^/(GP14^n^ + GP15^n^) × 1000* |
|  |  | ***GP^n^ = Ʃ(GP1^n^:GP15^n^)*** |
