# Supplementary material for: Glycosylation of Immunoglobulin G: Role of Genetic and Epigenetic Influences
Source: PLoS One. 2013 Dec 6;8(12):e82558. doi: 10.1371/journal.pone.0082558 (PMC3855797; doi:10.1371/journal.pone.0082558)
Supplement: Table S2 — List of glycans associated with circulating levels of triglycerides (log) and of C-reactive protein (Bonferroni P<7x10^-4^). (DOCX) [file pone.0082558.s002.docx]

**Table S2. List of glycans associated with circulating levels of triglycerides (log) and of C-reactive protein (Bonferroni *P*<7x10^-4^).**

| **Phenotype** | **Glycan** | **Beta[95%CI]** | **P** | ***h^2^*** |
| --- | --- | --- | --- | --- |
| tryglicerides | GP6 | 0.24[0.13,0.35] | 2.54 x10^-5^ | 0.75 |
| tryglicerides | GP6n | 0.24[0.13,0.35] | 3.30 x10^-5^ | 0.75 |
| tryglicerides | GP18 | -0.2[-0.32,-0.09] | 7.10 x10^-4^ | 0.73 |
| tryglicerides | FG2n/(BG2n + FBG2n) | -0.21[-0.33,-0.09] | 7.16 x10^-4^ | 0.69 |
| CRP | FG0n total/G0n | 0.01[0,0.01] | 3.09 x10^-4^ | 0.52 |
| CRP | GP8n | -0.01[-0.01,0] | 6.54 x10^-4^ | 0.80 |
| CRP | GP7n | -0.01[-0.01,0] | 6.81x10^-4^ | 0.73 |
