# Supplementary material for: Glycosylation of Immunoglobulin G: Role of Genetic and Epigenetic Influences
Source: PLoS One. 2013 Dec 6;8(12):e82558. doi: 10.1371/journal.pone.0082558 (PMC3855797; doi:10.1371/journal.pone.0082558)
Supplement: Table S3 — List of loci associated with all glycans with high heritability (h2>0.55). (DOCX) [file pone.0082558.s003.docx]

**Table S3. List of loci associated with all glycans with high heritability (*h^2^*>0.55).**

| **Glycan trait** | **Gene 1** | **Gene 2** | **Gene 3** | **Gene 4** |
| --- | --- | --- | --- | --- |
| GP1 | *SUV420H1* |  |  |  |
| GP2 | *IKZF1* | *ABCF2-SMARCD3* | *FUT8* |  |
| GP4 | *IL6ST-ANKRD55* |  |  |  |
| GP5 |  |  |  |  |
| GP6 | *ABCF2-SMARCD3* | *SYNGR1-TAB1-MGAT3-CACNA1I* |  |  |
| GP7 | *IKZF1* | *FUT8* |  |  |
| GP8 |  |  |  |  |
| GP9 |  |  |  |  |
| GP10 | *SMARCB1-DERL3* | *SYNGR1-TAB1-MGAT3-CACNA1I* |  |  |
| GP11 | *SMARCB1-DERL3* |  |  |  |
| GP12 | *FUT8* |  |  |  |
| GP13 |  |  |  |  |
| GP17 |  |  |  |  |
| GP18 | *ST6GAL1* | *B4GALT1* |  |  |
| FGS/(F+FG+FGS) | *ST6GAL1* | *B4GALT1* |  |  |
| FG2S2/(FG2+FG2S1+FG2S2) | *ST6GAL1* |  |  |  |
| FBS2/FS2 | *B4GALT1* | *SMARCB1-DERL3* | *SYNGR1-TAB1-MGAT3-CACNA1I* |  |
| FBS2/(FS2+FBS2) | *B4GALT1* | *SMARCB1-DERL3* | *SYNGR1-TAB1-MGAT3-CACNA1I* |  |
| GP1n | *SUV420H1* |  |  |  |
| GP2n | *IKZF1* | *ABCF2-SMARCD3* | *FUT8* |  |
| GP5n |  |  |  |  |
| GP6n | *ABCF2-SMARCD3* | *SYNGR1-TAB1-MGAT3-CACNA1I* |  |  |
| GP7n | *IKZF1* | *FUT8* |  |  |
| GP8n |  |  |  |  |
| GP9n |  |  |  |  |
| GP10n | *SMARCB1-DERL3* | *SYNGR1-TAB1-MGAT3-CACNA1I* |  |  |
| GP11n | *SMARCB1-DERL3* |  |  |  |
| GP12n | *FUT8* |  |  |  |
| GP13n |  |  |  |  |
| G0n | *IL6ST-ANKRD55* |  |  |  |
| G1n |  |  |  |  |
| Fn total | *IKZF1* | *FUT8* |  |  |
| FG0n total/G0n | *IKZF1* | *FUT8* |  |  |
| FG1n total/G1n | *IKZF1* | *FUT8* |  |  |
| FG2n total /G2n | *FUT8* |  |  |  |
| Fn | *IKZF1* | *SMARCB1-DERL3* | *SYNGR1-TAB1-MGAT3-CACNA1I* |  |
| FG0n/G0n | *IKZF1* | *FUT8* | *SMARCB1-DERL3* | *SYNGR1-TAB1-MGAT3-CACNA1I* |
| FG1n/G1n | *SMARCB1-DERL3* | *SYNGR1-TAB1-MGAT3-CACNA1I* |  |  |
| FG2n/G2n | *FUT8* |  |  |  |
| FBn | *SMARCB1-DERL3* | *SYNGR1-TAB1-MGAT3-CACNA1I* |  |  |
| FBG0n/G0n | *IKZF1* | *SMARCB1-DERL3* | *SYNGR1-TAB1-MGAT3-CACNA1I* |  |
| FBG1n/G1n | *SMARCB1-DERL3* | *SYNGR1-TAB1-MGAT3-CACNA1I* |  |  |
| FBG2n/G2n | *SMARCB1-DERL3* |  |  |  |
| FBn/Fn | *IKZF1* | *SMARCB1-DERL3* | *SYNGR1-TAB1-MGAT3-CACNA1I* |  |
| FBn/Fn total | *IKZF1* | *SMARCB1-DERL3* | *SYNGR1-TAB1-MGAT3-CACNA1I* |  |
| Fn/(Bn + FBn) | *IKZF1* | *SMARCB1-DERL3* | *SYNGR1-TAB1-MGAT3-CACNA1I* |  |
| Bn/(Fn + FBn) ‰ |  |  |  |  |
| FBG2n/FG2n | *SMARCB1-DERL3* |  |  |  |
| FBG2n /(FG2n + FBG2n ) | *SMARCB1-DERL3* |  |  |  |
| FG2n/(BG2n + FBG2n) | *SMARCB1-DERL3* |  |  |  |
| BG2n/(FG2n + FBG2n) ‰ |  |  |  |  |
